# An Integrative Review of the Military’s Approach to Talent Management: Insights for Cultivating Talent in Medical Education

**DOI:** 10.7759/cureus.102316

**Published:** 2026-01-26

**Authors:** Zachary R Smith, Teresa M Chan, Ajiri Ikede, Rakesh Patel, Christopher Stave, Margaret E Thornton, Stefanie S Sebok-Syer

**Affiliations:** 1 Emergency Medicine, Stanford University, Palo Alto, USA; 2 Emergency Medicine, Toronto Metropolitan University, Toronto, CAN; 3 McMaster Education Research Innovation and Theory (MERIT) Program, McMaster University, Hamilton, CAN; 4 Institute of Health Policy, Management, and Evaluation, Dalla Lana School of Public Health, University of Toronto, Toronto, CAN; 5 School of Medicine, The Chinese University of Hong Kong, Shenzhen, CHN; 6 Emergency Medicine, William Osler Health System, Brampton, CAN; 7 Family Medicine, Canadian Armed Forces, Ottawa, CAN; 8 Nephrology, Barts and The London School of Medicine and Dentistry, Queen Mary University of London, London, GBR; 9 Educational Leadership, Administration, and Research, Rowan University, Glassboro, USA; 10 Emergency Medicine, Stanford University Medical Center, Palo Alto, USA

**Keywords:** job alignment, medical education, military talent, talent assessment, talent management, workforce retention

## Abstract

Burnout remains a persistent challenge for the physician workforce. In this context, the ability to identify and retain physician talent plays a crucial role in optimizing workforce capacity. Other disciplines, such as the military, use comprehensive frameworks to identify, develop, and retain talent. These approaches may offer valuable insights for medical education, particularly with the increasing emphasis on competency-based training.

An integrative review examining the military’s approach to talent identification, development, facilitation, and management was conducted in 2024, with an updated search performed in October 2025. A total of 477 articles were identified and screened at the title and abstract level using predefined inclusion criteria. Twenty-one articles were ultimately included for data extraction.

Four major themes emerged: (1) the military defines talent holistically; (2) organizational structures align individual talent with specific positions to support well-being and maximize job performance; (3) individual talents are viewed as contributing to the success of a broader entity (e.g., a team, unit, or community); and (4) talent retention is a primary organizational focus.

Reviewing the military’s approach to identifying and retaining talent highlights the potential impact of a talent-focused framework in medical education. Addressing workforce challenges through a collective lens and aligning talent with specific roles may enhance physician well-being while also reducing burnout.

## Introduction and background

According to the American Medical Association, physician burnout remains a significant concern, with nearly 50% of physicians reporting symptoms of burnout. Despite modest improvements in recent years, two major contributors to burnout are job stress and a sense of being undervalued by one’s organization [1]. By placing greater emphasis on creating organizational structures that promote and support talent within medicine [2], we may enhance physicians’ sense of value, reduce burnout, and retain a more stable workforce. Currently, the concept of talent is broad and varies across fields and even within medical specialties. For the purposes of this paper, we define talent as individuals who demonstrate excellence or exceptional skills in a specific area. In medicine, individual talent is typically recognized during the application and admissions process, often through predetermined metrics or qualities set by admissions committees, but rarely beyond the recruitment phase of medical education. Physician training increasingly focuses on Competency-Based Medical Education (CBME), which emphasizes personalized learning, tailored progression, and outcome-based assessments. These assessments are often used to identify trainees who are not meeting expectations or milestones [3,4]. Typical methods for evaluating competence include (a) following the correct steps of a procedure [5], (b) auditing exposure to patient cases or specific pathologies [6], and (c) evaluating performance on predefined entrustable professional activities [7]. However, these assessments could be expanded or adapted to identify not only those who meet expectations but also those who exceed them. For example, measuring both the quantity and quality of ultrasound techniques and imaging could ensure trainees meet minimal competence while also flagging those with exceptional skill in this area. Although CBME is comprehensive and designed to tailor training to individual needs, it may inadvertently overlook high-potential individuals with specialized skills by focusing primarily on achieving minimal competence. By prioritizing baseline achievement, medical education risks missing outliers who demonstrate excellence or possess a niche talent that could be further developed. This highlights the need to evaluate our approach to identifying, developing, and retaining talent in medicine. Efforts to nurture talent should begin early in medical education and continue into independent practice.

Given that medical education currently lacks a well-defined approach for promoting and facilitating talent, this paper looks to an adjacent field, the military, to examine its practices around talent management. The military, with its extensive experience in recruitment, training, and workforce alignment to accomplish national defense objectives, offers valuable insights. This review represents a first step toward potentially adopting strategies from outside medicine to improve the medical training system. It is guided by the following questions: (1) How is talent identified and cultivated within the military context? (2) Which military approaches and strategies might be useful in recruiting, training, and retaining a more stable physician workforce? We found that the military relies on detailed frameworks to define individuals based on knowledge, skills, and behaviors, aligning talent with roles to improve both individual effectiveness and organizational performance, while also enhancing retention.

## Review

To identify insights on talent management in the military, we conducted an integrative review, which allowed us to summarize the available literature given the broad research questions. This approach enabled the integration of common themes and provided a foundation for future discussions on cultivating talent in medical education, without aiming to offer specific recommendations or guidelines [8]. To mitigate selection biases, we relied on well-established databases and placed no restrictions on publication dates. The initial search was conducted in July 2024 by one team member (CS) and updated in October 2025, using the following bibliographic databases and search engines: PubMed, Web of Science, ERIC, PsycInfo, Google, and Google Scholar. The exact search strategies for PubMed, Web of Science, ERIC, and PsycInfo are provided in Supplement 1. Searches using Google and Google Scholar were more iterative, incorporating terms related to the military (e.g., armed forces, army, naval, marines, air force, military, soldiers) and to talent (e.g., *aptitude*, *ability*, *expertise*, *flair*, *gift*).

The search yielded a total of 477 articles. These were uploaded to Covidence and screened by three reviewers (ZS, TMC, SSS) for relevance based on title and abstract. Discrepancies between reviewers were resolved through discussion and consensus, including voting to include or exclude articles. This process resulted in 47 articles being selected for full-text review. Articles were excluded if they focused solely on non-military populations (e.g., civilians); did not address talent development (e.g., focused only on retention via incentives); discussed only diversity, equity, and inclusion; or focused exclusively on workforce retention.

**Figure 1:**
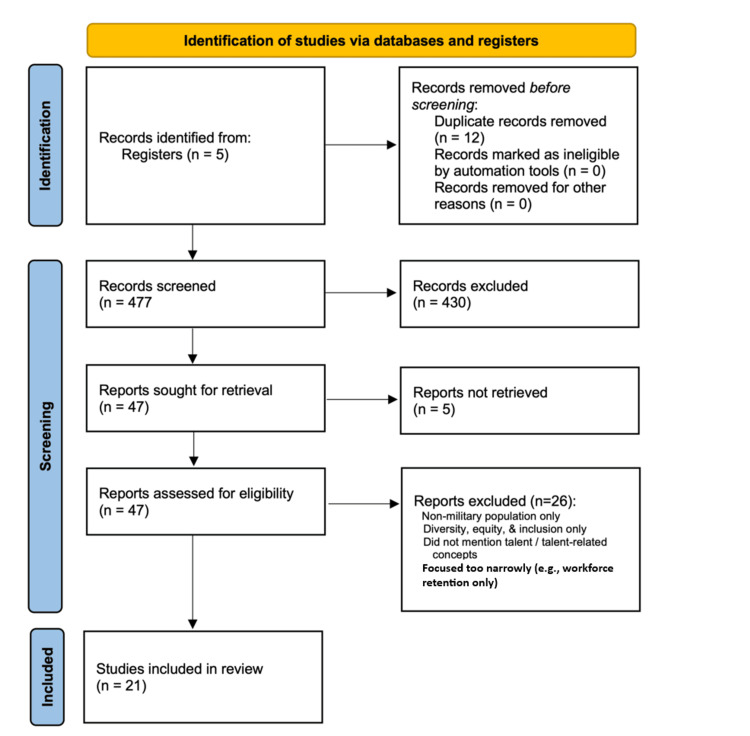
Flow diagram of the integrative review process for article selection.

Furthermore, five articles were excluded due to quality issues (e.g., no full text available), resulting in 21 relevant papers for data extraction (Figure 1). Most articles originated from North America, specifically the United States, and were published between 2017 and 2024. 

Results

The 21 articles [9-29] presented in Table 1 included a mix of study types: descriptive (14.3%), qualitative (4.8%), quantitative (14.3%), mixed-methods (14.3%), and others (52.4%). While most of the literature focused on the US military, we also identified studies from the Netherlands, Canada, Australia, and Brazil. Within the US military, the majority of studies examined the Army, with additional research on the Navy, Air Force, and Special Operations Units. 

**Table 1:** Summary of findings from the integrative scoping review of military-focused articles on talent assessment and development

Author (year)	Population	Framework	Themes
AlMamari and Traynor (2020) [21]	Airforce	No specific framework	Aptitude tests predict job performance
Forbes (2020) [27]	Navy, Army, Special Operations	Volatility, uncertainty, complexity, ambiguity (VUCA)	Team dynamics
Burrell et al. (2023) [22]	Army	No specific framework	Talent and data collection
Calleja et al. (2020) [23]	Army	No specific framework	Talent assessment and performance outcomes
Association of the United States Army (2021) [16]	Army	Army Talent Management Task Force, Assignment Satisfaction Key-Enlistment Marketplace (ASK-EM)	Talent and job alignment for NCOs
Association of the United States Army (2024) [17]	Army	Talent Alignment Program, Command Assessment Program (CAP), Integrated Personnel and Pay System (IPPS)	Talent and job alignment, talent marketplace
Chamberlin et al. (2020) [24]	Special Operations	Person-environment fit theory	Total Force Fitness (TFF), Capability-Based Blueprint (CBB), and Targeting System
US Army (2017) [10]	Army	Army People Strategy, Knowledge, Skills Behavior (KSBs), IPPS	Talent and data collection; career development
US Army (2021) [20]	Army, Army civilians	Army People Strategy	Career development, Master Training
Glerum and Royston (2023) [12]	Army	Army Talent Attribute Framework	Talent and data collection
Joo (2023) [11]	Army	Army Talent Management Task Force	Talent definition, Talent management, Retention, Talent and data collection
Keister (2019) [19]	Army	Talent Management Advisor Program	Talent management
Martin-Kowal et al. (2024) [25]	Airforce	No specific framework	Non-traditional talent assessment to predict job performance
Griffin and McClary (2015) [13]	Army	No specific framework	Talent management; Talent and job alignment; Talent and data collection
Miranda et al. (2019) [14]	Military	No specific framework	Talent and data collection
Nissen and Tick (2018) [15]	Navy	No specific framework	Talent management, Retention, Talent definition
Oprins et al. (2018) [26]	Military and civilian	Person-environment fit theory	Adaptability competency
Roberson et al. (2022) [9]	Army, Special Operations	Army People Strategy, Army Talent Management Task Force, Talent Management Profile System	Talent and data collection
Rodden-Aubut and Tracey (2022) [28]	Military	Person-environment fit theory	Aptitude tests predict job performance
Association of the United States Army (2019) [18]	Army	Integrated personnel and pay system, Berens' 4 temperaments	Team dynamics
Spain et al. (2020) [29]	Army	Person-environment fit theory	Person-environment fit theory

Four major themes emerged from the review: (1) the military defines talent holistically and meticulously; (2) organizational structures align individual talent with job positions to support well-being and maximize performance; (3) individual talents are viewed as contributing to the success of a broader entity (e.g., team, unit, or community); and (4) retaining talent is a primary focus within the military. While medicine has focused on retaining physicians, the concept of aligning individuals’ innate talents with specific roles and considering their contributions within larger health systems is relatively novel.

The articles offered a general sense of how the military defines talent. In practice, the military uses a data-driven approach to identify a soldier’s or officer’s talent, often through the knowledge, skills, and behavior (KSB) or knowledge, skills, attributes, and observations (KSAO) models [9-14]. No single, consistent definition of talent emerged; instead, the literature described a detailed system for assessing a service member’s characteristics or unique traits that could be interpreted as relevant talent for specific roles or assignments. For example, the Army Talent Attribute Framework uses a three-tiered system to qualify individuals based on their KSAOs [12]. Under disposition (Tier 1) and military-specific traits (Tier 2), a soldier’s warrior ethos can be assessed (Tier 3). Similarly, under expertise and personal competence (Tier 1) and mechanical/technological fluency (Tier 2), advanced computer skills can be rated (Tier 3). Only two articles framed talent differently: one defined it as possessing certain personal attributes, such as being “highly motivated, humble, caring, and adaptable,” while another measured it as competency in a specific sector (e.g., information technology) relative to rank [15].

Several articles highlighted the theme of talent-job alignment, matching service members to positions based on their knowledge, skills, and attributes [13,16,17]. These studies described a shift from a system where assignments were earned through promotion or filling workforce gaps to a “job marketplace” approach, in which the military prioritizes the soldier’s expressed interests and talents [16]. Examples of frameworks supporting this transformation include the Command Assessment Program, Army Talent Alignment Process, Army Talent Attribute Framework, and Integrated Personnel and Pay System [9-12,16-18]. These systems consolidate employee data based on KSBs to better align individuals with roles and leadership opportunities [10]. The identification of top talent is an ongoing area of focus and scholarship within the military [22-25,29].

Additionally, several articles addressed talent management [11-13,15,19], including the role of career development [10,20] and mastery training [20]. The theme of team dynamics, considering individual talents in the context of team success, was also common [18,30]. Finally, although studies focusing primarily on retention were excluded, multiple articles still examined talent in relation to maintaining a skilled and effective workforce [2,11].

Discussion

Sectors outside of healthcare can provide valuable insights for improving methods of talent cultivation, which may help reduce provider burnout and support the recruitment, training, and retention of a stable workforce. Our review of the military’s talent management systems revealed a multidimensional approach to collecting talent-related data and fostering individual abilities. It highlighted how the military balances the needs of individuals with those of the team or unit, and how talent development contributes to workforce retention efforts. 

*Talent Identification and Cultivation*****

The article by Joo provides valuable insight into the individual soldier’s perception of talent, defining it as “individual-driven/derived experience, qualities, and skillset” [11]. Organizationally, the military casts a wide net to capture all types of talent among its soldiers using KSB or KSAO data. This can be compared with the holistic assessment of medical trainees entering medical school or residency, where the focus has shifted from grades and test scores to the Experiences, Attributes, and Metrics (EAM) framework outlined by the Association of American Medical Colleges (AAMC) in Holistic Considerations for the Admission Cycle [30-32]. In this framework, experiences are defined as “life circumstances and chosen activities,” attributes as “skills, abilities, personal qualities, core competencies… and relevant demographic identities,” and metrics as “quantitative components of an applicant’s portfolio” [31]. Although the AAMC’s primary goal was to improve diversity, the framework also reflects a broader shift toward valuing non-academic traits [31].

Both approaches, medical trainee EAM data and a soldier’s KSB or KSAO information, aim to identify talent by highlighting previously overlooked qualities. However, the military’s system appears to qualify these traits more comprehensively and precisely. Military talent assessment is also closely linked to career progression; a soldier’s current assignment may provide experiences or skills, identified through the three-tiered Army Talent Attribute Framework, which are relevant to the next position [12]. In contrast, a medical trainee’s talent assessment is largely momentary, focused on admission to medical school or residency. Trainees are typically tied to one institution for several years and do not experience the frequent role transitions seen in military service. Implementing a longitudinal, holistic evaluation system in medicine could better cultivate individual talent. Recognizing the unique skills and abilities of residents or attending physicians may also strengthen their sense of value within an organization, which is associated with reduced burnout [33]. 

*Talent, Job Alignment, and Looking Beyond the Individual*****

Our second research question focused on identifying military strategies that could inform the recruitment, training, and retention of a stable physician workforce. The AAMC emphasizes that a trainee’s qualities are evaluated for how they “contribute to the program’s unique goals, learning environment, and the practice of medicine” [21,31-33]. In contrast, the military evaluates a soldier’s value within the context of the team or unit [16,18,30]. While medicine tends to view individuals as contributing to the broader organization or field, the military emphasizes the individual’s ability to enhance team success. One article applied psychologist Dr. Linda V. Berens’ four temperaments model to compare military officers with positions on an American football team (e.g., quarterback, running back) to facilitate job placement and strengthen team cohesion [18]. Using this approach, medical education could reconsider how a trainee’s skills, knowledge, and attributes are integrated into the clinical team. Longitudinally managing a learner’s talents in this way could improve job satisfaction and retention, as physicians report nearly a 40% reduction in burnout when teamwork is a positive experience [33].

Beyond team dynamics, other relationships remain relevant after training. At an international conference, attendees noted the role of the broader community in physician job placement. Viewing a physician’s talents in relation to the needs of the populations they serve could be valuable. For example, a trainee’s linguistic skills or cultural competence may be particularly useful in certain regions [34]. Currently, systems like the National Resident Matching Program (“Match”) align students and residency programs based on mutual preferences [35]. Expanding the focus from talent purely in recruitment to talent in job or community alignment could enhance workforce stability and better utilize individual strengths. 

Retention

Although our integrative review excluded articles focused solely on retention, we included studies that considered retention in the context of talent management. For example, an interview with former Army Deputy Chief of Staff Lt. Gen. James McConville highlighted the importance of evaluating an individual’s long-term goals and identifying their talent profile to align them with appropriate roles, thereby enhancing job satisfaction [10]. This approach can help retain soldiers who wish to remain in the military, benefiting both the individual and the organization, whether at the strategic level or within smaller, local units. Retention is a significant concern in the military, where only 10%-30% of US Army service members remain until retirement. In contrast, attrition in US medical education is relatively low: fewer than 5% of medical students leave medical school, and 1%-26% of graduate medical education trainees exit depending on the specialty [10,36,37]. Military strategies for training and retention may be most relevant to medicine after initial undergraduate and residency training, during physicians’ ongoing careers and continuing professional development. Applying these approaches could help maintain a stable physician workforce, reduce burnout, and provide a foundation for future research. 

*Implications for Medical Education*****

We have focused on ways in which medical education might learn from the military’s approach to talent management, with potential implications for addressing physician burnout. According to the 2023 American Medical Association Organizational Biopsy, 48.2% of respondents report at least one symptom of burnout, and only 50% feel valued as a physician [1]. Burnout is significantly associated with lower patient satisfaction scores, higher rates of physicians being named in malpractice suits, and increased organizational costs due to higher turnover and referral rates [1,38]. Addressing physician burnout is complex, but one potential area of focus is how providers perceive that their unique skills and attributes are recognized. Currently, individual talent is seldom considered beyond medical school admissions or residency matching. Yet, cultivating potential, developing skills, and providing coaching can effectively mitigate burnout [39,40].

Medical residency programs that offer ample elective time allow for talent exploration and growth. Coupled with effective mentorship, this may help align trainees with careers where they feel valued, are more likely to be retained, and experience less burnout. The military also offers insights regarding team dynamics. Medical training often emphasizes individual achievement, as applicants are assessed on academics, standardized testing, and performance in clerkships and residency. The concept of the trainee as part of a larger system, functioning within a team or serving a community, is often overlooked. Yet, physicians operate within healthcare teams when delivering patient care, and effective teamwork is critical for both patient outcomes and professional well-being. Team-based training programs, such as Strategies and Tools to Enhance Performance and Patient Safety (TeamSTEPPS), have already demonstrated improvements in patient safety culture [41,42].

Limitations

Our review has several limitations. First, the predominance of US Army-based articles may introduce bias, which should be considered when applying military lessons to medical education. Second, many military talent development strategies are framed primarily through the lens of retention, which may limit their applicability to medicine, where retention is less of a concern. Several articles describe the implementation of large-scale processes to assess talent and align individuals with roles, but we found little empirical data evaluating the outcomes of these initiatives, which further limits the transferability of these approaches. Finally, the absence of a clear, comprehensive definition of talent in both the military and medical contexts constrains our ability to conceptualize the construct. Without a standardized definition, rigorous methods for measuring talent are lacking, limiting generalizability and applicability across disciplines. 

Future research

Although our broad literature search identified only 21 relevant papers, we were still able to gain insights into the military’s processes for holistic talent assessment, development, and aligning employees with roles suited to their skills and abilities. Members of our team previously conducted a scoping review in medicine and health professions education and found even fewer frameworks and theories addressing talent in the medical education literature [43]. Future empirical studies could evaluate the effectiveness of these strategies when adapted to medical education, potentially through pilot programs focused on talent development. Data on how such programs impact provider burnout, enhance team dynamics, and improve patient care would be valuable, ideally accompanied by published outcomes from the military’s talent assessment and job alignment initiatives. Additionally, such studies could illuminate cultural differences between the military and healthcare institutions that might influence implementation, as well as potential variation in success across medical specialties. This information could help justify the adaptation of these talent management tools to healthcare settings. 

## Conclusions

To examine the military’s approaches to talent identification and development, we reviewed the existing literature and identified several recurring themes. These included a detailed framework for defining individuals based on knowledge, skills, and behaviors; aligning these traits with specific roles; and viewing individual talents in the context of team function and workforce retention. Although the review did not identify studies reporting outcomes of military talent interventions or robust data on job satisfaction following their implementation, this work represents an important first step. Incorporating more holistic assessments of trainees’ skills and interests, aligning these with potential career pathways, emphasizing team-based training, and continuing to explore external talent cultivation strategies may help medical education reduce physician burnout and support workforce retention.

## Appendices

Supplement 1: search strategy

ERIC

(MAINSUBJECT.EXACT.EXPLODE("Military Personnel") OR title(army OR military OR "armed force*" OR navy OR "air force" OR marines OR army)) AND (MAINSUBJECT.EXACT.EXPLODE("Talent") OR MAINSUBJECT.EXACT.EXPLODE("Talent Development") OR title(skill OR "ability" OR "aptitude" OR "capability" OR "competence" OR "expertise" OR "flair" OR "gift" OR "knack" OR "prowess"))

*PubMed*
** **

("Aptitude"[Mesh] OR "skill" [ti] OR "ability" [ti] OR "aptitude" [ti] OR "capability" [ti] OR "competence" [ti] OR "expertise" [ti] OR "flair" [ti] OR "gift" [ti] OR "knack" [ti] OR "prowess" [ti]) AND ("Military Personnel"[Mesh] OR army [ti] OR military [ti] OR "armed force*" [ti] OR navy [ti] OR "air force" [ti] OR marines [ti] OR army [ti]) AND English [lang] AND 2015:2025 [dp] NOT letter [pt]

Web of Science

TI=("skill" OR "ability" OR "aptitude" OR "capability" OR "competence" OR "expertise" OR "flair" OR "gift" OR "knack" OR "prowess") AND TI=(army OR military OR “armed force*” OR navy OR “air force” OR marines OR army) limited to English, peer review journal articles, and 2015-present

*PsycInfo*
**  **

title(skill" OR "ability" OR "aptitude" OR "capability" OR "competence" OR "expertise" OR "flair" OR "gift" OR "knack" OR "prowess") OR subject("Ability ") AND  MAINSUBJECT.EXACT.EXPLODE("Army Personnel") OR title(army OR military OR “armed force*” OR navy OR “air force” OR marines OR army) limited to English, peer review journal articles, and 2015-present
